# Nallan’s Direct Ray: An Innovative Gyroscopic-Guided Radiographic Device for Intraoral Radiography

**DOI:** 10.3390/diagnostics16030386

**Published:** 2026-01-25

**Authors:** Nallan C. S. K. Chaitanya, Nada Tawfig Hashim, Vivek Padmanabhan, Riham Mohammed, Sharifa Jameel Hossain, Sadiah Fathima, Nurain Mohammad Hisham, Neeharika Satya Jyothi Allam, Shishir Ram Shetty, Rajanikanth Yarram, Muhammed Mustahsen Rahman

**Affiliations:** 1 Department of Oral Medicine and Radiology, RAK College of Dental Sciences, RAK Medical & Health Sciences University, Ras-AlKhaimah 12973, United Arab Emirates; 2 Department of Periodontics, RAK College of Dental Sciences, RAK Medical & Health Sciences University, Ras-AlKhaimah 12973, United Arab Emirates; 3 Department of Pediatric and Preventive Dentistry, RAK College of Dental Sciences, RAK Medical & Health Sciences University, Ras-AlKhaimah 12973, United Arab Emirates; 4 Department of Oral Surgery, RAK College of Dental Sciences, RAK Medical & Health Sciences University, Ras-AlKhaimah 12973, United Arab Emirates; 5 RAK College of Dental Sciences, RAK Medical & Health Sciences University, Ras-AlKhaimah 12973, United Arab Emirates; 6 Department of Oral Medicine, Tatva Dental Clinic, Hyderabad 500060, India; 7 Department of Oral and Craniofacial Health Sciences, College of Dental Medicine, University of Sharjah, Sharjah 27272, United Arab Emirates; 8 General Surgery, Life Line Hospitals, Abu Dhabi 94666, United Arab Emirates

**Keywords:** gyroscopic guidance, laser alignment, intraoral radiography, geometric accuracy, dental imaging, radiographic errors, diagnostic precision

## Abstract

**Background**: Intraoral radiography remains highly operator-dependent, with small deviations in beam angulation or receptor placement leading to geometric distortions, diagnostic inaccuracies, and repeated exposures. This pilot study introduces and evaluates a gyroscopic-guided, laser-assisted radiographic device designed to standardize cone positioning and improve the geometric reliability of bisecting-angle intraoral radiographs. **Methods**: Eighteen dental graduates and practitioners performed periapical radiographs on phantom models using a charge-coupled device (CCD) sensor over six months. Each participant obtained six standardized projections with and without the device, yielding 200 analysable radiographs. Radiographic linear measurements included tooth height (occluso–apical dimension) and tooth width (mesio-distal diameter), which were compared with reference values obtained using the paralleling technique. Radiographic errors—including cone cut, elongation, proximal overlap, sliding occlusal plane deviation, and apical cut—were recorded and compared between groups. **Results**: Use of the gyroscopic-guided device significantly enhanced geometric accuracy. Height measurements showed a strong correlation with reference values in the device group (r = 0.942; R^2^ = 0.887) compared with the non-device technique (r = 0.767; R^2^ = 0.589; *p* < 0.0001). Width measurements demonstrated similar improvement (device: r = 0.878; R^2^ = 0.770; non-device: r = 0.748; R^2^ = 0.560; *p* < 0.0001). Overall, the device reduced technical radiographic errors by approximately 62.5%, with significant reductions in cone cut, elongation, proximal overlap, sliding occlusal plane errors, and tooth-centering deviations. **Conclusions**: Integrating gyroscopic stabilization with laser trajectory guidance substantially improves the geometric fidelity, reproducibility, and diagnostic quality of intraoral radiographs. By minimizing operator-dependent variability, this innovation has the potential to reduce repeat exposures and enhance clinical diagnostics. Further clinical trials are recommended to validate performance in patient-based settings.

## 1. Background

Intraoral radiography plays an indispensable role in dental diagnosis by providing detailed visualization of teeth and their supporting structures while maintaining minimal radiation exposure to patients [1,2]. Despite its clinical importance, obtaining optimal image quality remains a significant challenge, largely due to technical inaccuracies in sensor positioning and improper alignment of the X-ray beam [3]. Even minor deviations in horizontal or vertical angulation can result in distortions such as elongation or foreshortening, which compromise diagnostic accuracy and frequently necessitate repeated exposures [4]. These repeated attempts not only increase the cumulative radiation dose but also reduce clinical efficiency and delay patient management [5]. Achieving correct film or sensor placement, as well as precise beam angulation, constitutes the core difficulty in intraoral radiography. These challenges are especially pronounced when operators lack adequate experience or familiarity with radiographic projection geometry, making the technique highly operator-dependent and prone to error [6,7].

Conventional intraoral radiographic techniques require deliberate and coordinated control of both horizontal and vertical angulations, in addition to ensuring that the intraoral receptor is positioned parallel to the long axis of the tooth or appropriately placed within the bisecting-angle geometry [6,8]. For both dental practitioners and undergraduate students, this multi-step requirement often presents difficulties, resulting in inconsistent image quality and the frequent need for repeat radiographs to achieve diagnostically acceptable images [3]. This operator dependence continues to be a major limitation in maintaining uniform standards of radiographic accuracy across clinical settings [9].

Recent technological advancements provide promising avenues to address these long-standing challenges. Gyroscopic technology, widely used in aerospace navigation and consumer electronics for stabilization and orientation detection, offers potential adaptability for dental radiography. Likewise, laser guidance systems have demonstrated high precision in dental procedures and have been successfully integrated into panoramic imaging to ensure accurate patient positioning within the focal trough [10]. However, their application to intraoral radiographic procedures remains limited, despite the clear potential to enhance alignment accuracy and reduce positioning errors [10].

**Figure 1:**
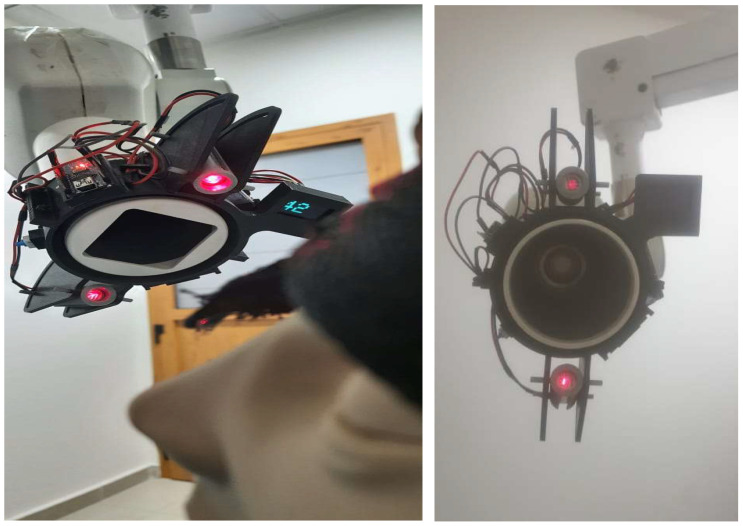
Nallan’s Direct Ray gyroscopic-guided intraoral radiographic device shown in two views during positioning. The red dots represent active status LEDs used for alignment and stabilization feedback. The green element corresponds to the digital display indicating device operating parameters.

The present pilot study was designed to integrate gyroscopic orientation and laser-based visualization into routine intraoral radiography, addressing common errors in beam angulation and receptor placement. The newly developed device, a gyroscopic-guided and laser-enabled model mounted directly onto the cone of a standard dental X-ray machine (Figure 1), provides real-time feedback on beam orientation in relation to sagittal and coronal planes. This dual-modality guidance aims to enhance the accuracy of vertical angulation, assist practitioners in locating anatomical reference points, and ultimately ensure projection parameters that yield geometrically accurate images. By providing continuous visualization of the X-ray beam trajectory and stabilizing angular alignment through gyroscopic feedback, the device is designed to minimize distortions such as elongation and foreshortening and reduce the overall rate of technical errors. Consistent and accurate image acquisition could subsequently decrease the need for repeated exposures, thereby improving diagnostic efficiency and patient safety.

In this context, the objectives of the study are threefold: to assess the geometric accuracy of intraoral radiographic images produced using the traditional bisecting-angle technique without gyroscopic guidance; to evaluate the accuracy and reproducibility of images obtained using the newly developed gyroscope-guided, laser-assisted device; and to compare both methods in terms of their ability to reduce radiographic distortions and technical errors.

The need for this study is strongly justified by the persistent limitations of conventional intraoral radiography, which remains highly operator-dependent and susceptible to avoidable errors that compromise diagnostic precision [11,12]. Despite major advances in digital sensor technology, the fundamental issue of achieving correct angulation and consistent image geometry has remained largely unaddressed [13]. By introducing a device capable of guiding operators in real time, stabilizing beam orientation, and reducing geometric distortions, this study not only fills a critical gap in current radiographic practice but also contributes to improving patient safety by lowering the frequency of repeat exposures. This research therefore responds to an urgent clinical need for more reproducible, accurate, and operator-independent intraoral radiographic techniques.

## 2. Materials and Methods

### 2.1. Study Design

The present pilot study was designed to evaluate the effectiveness of a gyroscopic-guided radiographic device in enhancing the geometric accuracy of intraoral radiographs. This prospective, single-centre, crossover study was conducted over a six-month period in the dental radiology clinic of RAK College of Dental Sciences (RAKCODS) using a controlled, laboratory-based phantom model setup. Ethical approval for this study was obtained from the Institutional Review Board of RAK Medical & Health Sciences University prior to initiation of data collection (Approval No. RAKMHSU-REC-025-2-22/23-F-D, approval date: 10 November 2022). All participants provided informed consent before enrolment.

### 2.2. Participants and Study Design

Eligible participants included dental graduates and general practitioners with prior experience in intraoral radiographic techniques, ensuring that the study outcomes were not influenced by basic operator inexperience. Dental specialists and undergraduate students were excluded to maintain homogeneity in operator proficiency. Participants were randomly allocated into two groups (Group A and Group B), each consisting of nine operators. A crossover design was employed so that every participant acted as his or her own control, thereby reducing inter-operator variability. Each participant performed a minimum of six radiographic projections, resulting in three maxillary and three mandibular images composed of one anterior and two posterior views for each arch.

A randomized crossover design was employed. Participants were randomly assigned to one of two sequences (Group A or Group B) using a simple random allocation procedure. Group A performed radiographic acquisitions first using the non-device (ND) technique followed by the device-assisted (D) technique, whereas Group B followed the reverse sequence, starting with the device-assisted technique and then switching to the non-device technique. This counterbalanced design ensured that each participant served as their own control and minimized potential order-related bias.

To control for operator learning and carryover effects, all participants underwent a standardized familiarization session with both techniques prior to data collection. In addition, a wash-in period consisting of trial acquisitions was allowed before each technique, and images obtained during these trial runs were excluded from analysis. The use of counterbalanced sequences and within-operator comparisons further reduced the influence of learning effects on the study outcomes.

### 2.3. Device Fabrication and Technical Specifications

The gyroscopic-guided radiographic system evaluated in this study, designated Nallan’s Direct Ray (NDR), Version 1.0, was custom-fabricated as an external, cone-mounted guidance module that provides real-time angular orientation feedback during intraoral radiographic acquisition. The system integrates a microelectromechanical system (MEMS)-based gyroscopic sensor, a microcontroller-based processing unit, and a visual laser projection module, all housed within a compact and lightweight casing (Figure 1). The laser projection unit used in the device was a low-power visible red laser classified as Class 2 according to IEC 60825-1, ensuring safe use under normal operating conditions [14]. The laser beam exhibited minimal divergence over the short working distances relevant to intraoral radiography, producing a stable and clearly visible crosshair pattern on the target surface.

Laser alignment accuracy was ensured through mechanical co-alignment of the laser module with the gyroscopic sensor axes during device assembly. The projected crosshair was calibrated to represent the sagittal and coronal reference planes corresponding to the cone’s central axis. During calibration, the X-ray cone was positioned in a neutral, perpendicular orientation, and the laser projection was adjusted to intersect symmetrically over predefined reference points. This alignment protocol was verified at the beginning of each imaging session to maintain consistent projection accuracy.

A tri-axial MEMS gyroscopic sensor (MPU-6050; InvenSense, San Jose, CA, USA), incorporating both a gyroscope and an accelerometer, was used to detect angular displacement of the X-ray cone along the sagittal and coronal planes. This sensor was selected for its compact size, high angular resolution, and stable performance in low-vibration environments. The gyroscopic unit was rigidly mounted within the device housing and mechanically aligned with the longitudinal axis of the X-ray cone to ensure accurate correspondence between cone orientation and sensor output.

Angular data acquired by the sensor were transmitted to an onboard microcontroller (Arduino Nano; Arduino AG, Somerville, MA, USA), which processed raw signals in real time. A sensor fusion algorithm combining gyroscope and accelerometer data was implemented to calculate cone orientation relative to the gravitational vertical reference. The processed angular deviations were continuously translated into corresponding visual cues through an integrated laser projection system.

**Figure 2:**
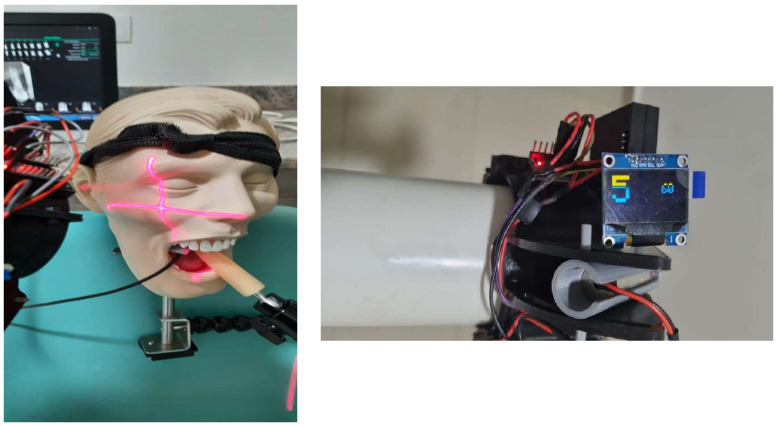
Nallan’s Direct Ray gyroscopic-guided intraoral radiographic device demonstrated on a dental phantom head and shown in close-up view. The projected red laser lines indicate the alignment axis for beam positioning, while the red LEDs represent active device status and stabilization feedback. The colored numeric display corresponds to real-time operating parameters of the device.

The visual output consisted of a crosshair-shaped laser pattern (“+”) projected onto the patient or phantom surface, representing the sagittal and coronal reference planes. Correct cone alignment was indicated when the projected laser lines intersected symmetrically over predefined anatomical reference points. Deviations from the optimal angulation resulted in visible displacement of the laser crosshair, providing immediate visual feedback to the operator and enabling real-time correction prior to image acquisition (Figure 2).

The device was affixed to the cylindrical surface of the X-ray cone using a custom-designed, non-invasive mounting bracket that allowed secure attachment without modification of the original radiographic equipment. The mounting system ensured fixed spatial alignment between the sensor axes and the cone while preventing rotational drift during routine handling. The device was positioned so as not to interfere with cone movement, patient positioning, or radiation emission.

The fully assembled prototype had an approximate total weight of 85 g, allowing stable attachment without affecting cone balance or maneuverability. Power was supplied by a rechargeable lithium-ion battery, providing sufficient capacity for multiple imaging sessions. The prototype was fabricated using commercially available electronic components and a custom mounting bracket, with an estimated material cost of approximately USD 120–150, excluding development time.

Prior to data collection, the device underwent calibration to establish baseline angular reference values. Calibration was performed by positioning the X-ray cone perpendicular to the floor and aligning it with standardized reference planes, after which the gyroscopic sensor was zeroed. This calibration procedure was repeated at the beginning of each imaging session to ensure consistent performance and to compensate for potential sensor drift.

Prior to formal data collection, all operators participated in a standardized familiarization session with the gyroscopic-guided device. This training phase consisted of a brief orientation to the device components, visual feedback mechanism, and calibration procedure, followed by supervised trial radiographic acquisitions on phantom models. These trial images were not included in the final analysis. The purpose of this learning phase was to ensure basic operational proficiency with the device and to minimize skill-related bias during the study period.

According to the manufacturer’s specifications, the MPU-6050 tri-axial MEMS sensor provides an angular resolution of approximately 0.01–0.02° with a typical sensitivity of 131 LSB/°/s at the selected full-scale range. Under static or low-vibration conditions, such as those encountered during intraoral radiographic positioning, the sensor demonstrates stable short-term angular measurements with minimal drift. The calibration procedure described above was used to align the sensor output with a gravitational reference and to compensate for any baseline offset, thereby ensuring reproducible angular feedback across imaging sessions.

### 2.4. Radiographic Procedure

Radiographic procedures were conducted using a standard intraoral dental X-ray unit equipped with a Charge-Coupled Device (CCD) sensor. In the first phase of the crossover, operators in Group A performed radiographs using the conventional bisecting-angle technique without the gyroscopic-guided device. In the second phase, the same participants performed identical projections using the gyroscopic-guided, laser-enabled device mounted onto the X-ray tube head. The device projected a crosshair-shaped laser pattern (“+”), corresponding to the sagittal and coronal planes, alongside vertical angulation indicators specific to the intended tooth region. This allowed real-time visualization of beam alignment relative to anatomical reference points, enabling more precise angulation during exposure (Figure 2).

To ensure methodological rigor, the X-ray machine output, exposure parameters, and focus-to-skin distance were standardized for all projections. The phantom model was stabilized using a fixed dental apparatus to eliminate variability in head orientation or occlusal plane positioning. Prior to commencing the study, the gyroscopic device underwent calibration according to the manufacturer’s technical specifications to verify accurate angular feedback. Calibration was repeated at the beginning of every session to ensure consistency. Additionally, reproducibility checks were performed by acquiring test radiographs at predetermined angulations to confirm device stability throughout data collection.

A minimum sample size of 200 intraoral radiographs was determined to be adequate for generating preliminary conclusions, allowing for correlation analyses and meaningful comparisons between device-assisted and non-assisted techniques. Accordingly, a total of 200 intraoral radiographs were acquired and deemed analysable. Subsets of these images were subsequently included in specific analyses based on predefined eligibility criteria for linear measurements and radiographic error assessment.

A sample size of 200 radiographs was considered adequate to detect moderate-to-strong correlations (r ≥ 0.70) and within-participant differences in linear measurements with acceptable precision, while also allowing meaningful comparison of error frequencies between acquisition techniques. Given the pilot nature of the study and the crossover design, this sample size was deemed appropriate for exploratory evaluation of device performance.

All radiographs were digitally stored and coded to mask both the acquisition technique and operator identity. Image evaluation was performed by a blinded, experienced oral radiologist to ensure objective and consistent assessment.

### 2.5. Image Evaluation and Error Assessment

Radiographic errors were defined operationally prior to image evaluation to ensure consistency and reproducibility. Cone cut was defined as incomplete receptor coverage resulting from misalignment between the X-ray beam and the image receptor. Elongation referred to an apparent increase in tooth height caused by insufficient vertical angulation, whereas foreshortening was defined as apparent shortening of the tooth resulting from excessive vertical angulation. Proximal overlap was defined as superimposition of adjacent proximal tooth surfaces due to incorrect horizontal angulation. Apical cut referred to incomplete visualization of the apical region caused by improper receptor placement or beam positioning. Blur was defined as loss of image sharpness attributable to motion during exposure.

Sliding occlusal plane error was defined as distortion resulting from improper alignment of the image receptor or X-ray cone relative to the occlusal plane, leading to apparent tilting or displacement of the occlusal reference line within the radiographic image. Tooth not centered referred to off-centre positioning of the tooth of interest within the radiographic field, resulting in incomplete or asymmetric visualization.

Geometric measurements, particularly the radiographic height and width of selected teeth, were obtained from reference images produced using the paralleling technique. These reference values served as the gold standard for comparison.

For each radiograph acquired using the bisecting-angle technique, with and without the device, discrepancies in tooth height and width were measured by an experienced radiologist to assess geometric distortion. Radiographs with incomplete visualization of the anatomical landmarks required for specific measurements were excluded on a per-analysis basis. As a result, different subsets of the total dataset were used for individual analyses. Of the total 196 radiographs included for error assessment, 140 radiographs met the predefined eligibility criteria for tooth height measurements, while 98 radiographs met the criteria for tooth width measurements. These exclusions were primarily due to partial apical cut-off, proximal overlap, or off-centering that precluded reliable linear measurement. The corresponding sample sizes are reported separately for each analysis to ensure transparency. In addition, each participant completed a standardized data sheet documenting perceived difficulties, procedural deviations, and any technical errors encountered during image acquisition, including cone cut, proximal overlap, elongation, foreshortening, sliding occlusal plane deviation, apical cut, blur, and failure to center the tooth of interest. Operator-reported errors were cross-checked with radiologist assessments to ensure consistency and reliability.

Comparisons between the two techniques were conducted to assess differences in geometric accuracy, reproducibility, and radiographic error frequency.

### 2.6. Data Analysis Plan

Statistical analyses were performed using Microsoft Excel (Microsoft Corporation, Redmond, WA, USA) and GraphPad Prism version 9.5.1 (GraphPad Software, San Diego, CA, USA). Continuous variables were summarized as mean ± SD, while categorical variables were expressed as frequencies and percentages. Comparisons between device-assisted and non-device techniques for continuous measurements were conducted using paired *t*-tests, reflecting the crossover study design in which each participant served as their own control.

The strength of association between radiographic measurements and reference values was assessed using Karl Pearson’s correlation coefficient, with coefficients of determination (R^2^) also reported. A *p*-value of less than 0.05 was considered statistically significant.

Tooth length and breadth measurements obtained with and without the device were analyzed separately based on predefined eligibility criteria, resulting in variable sample sizes across analyses.

### 2.7. Statistical Power and Sample Size Considerations

This study was designed as a pilot investigation to explore the feasibility, technical performance, and preliminary effectiveness of a gyroscopic-guided radiographic device. As such, a formal a priori sample size calculation was not performed. Instead, the sample size was determined pragmatically to allow meaningful estimation of effect sizes, variability, and error patterns under controlled phantom conditions, which are essential parameters for planning future confirmatory trials.

To provide additional transparency regarding the adequacy of the achieved sample size, a post hoc power analysis was conducted based on the observed effect sizes and final sample sizes. For tooth height measurements, the device-assisted technique demonstrated a strong correlation with the reference standard (r = 0.942, *n* = 140). At a two-sided significance level of α = 0.05, this corresponds to an achieved statistical power of >99%. Similarly, for tooth width measurements, the observed correlation (r = 0.878, *n* = 98) also yielded an achieved power of >99%.

These post hoc estimates indicate that the present study was sufficiently powered to detect the observed effects. However, consistent with current statistical recommendations, post hoc power calculations are reported for descriptive purposes only and should not be interpreted as a substitute for prospective sample size planning. The primary objective of this pilot study was to generate reliable effect size estimates and variance parameters to inform the design of future adequately powered, patient-based clinical trials.

## 3. Results

### 3.1. Tooth Length Measurements

Of the total analyzable radiographs, 140 images per group met the criteria for accurate tooth height measurement and were included in the analysis.

**Figure 3:**
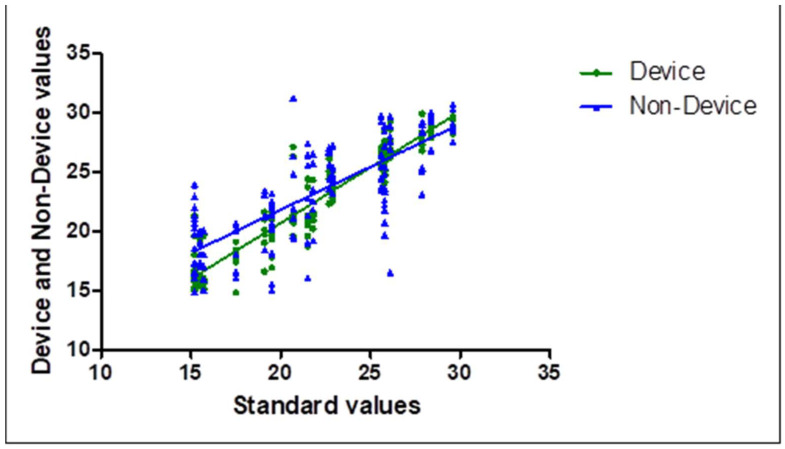
Scatter plot illustrating the correlation between tooth height measurements obtained using device-assisted and non-device techniques.

Correlation analysis of radiographic tooth height measurements obtained using the device-assisted and non-device techniques is presented in Table 1. Statistically significant correlations with reference measurements obtained using the paralleling technique were observed for both techniques (*p* < 0.0001). The relationship between measured and reference tooth height is illustrated in Figure 3.

### 3.2. Tooth Width Measurements

A subset of 98 radiographs per group fulfilled the criteria for tooth width measurement and comprehensive radiographic error evaluation and was included in these analyses.

**Figure 4:**
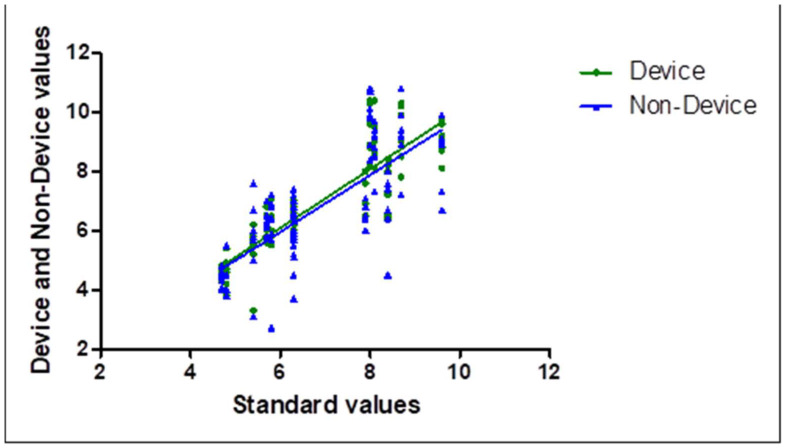
Scatter plot illustrating the correlation between tooth width measurements obtained using device-assisted and non-device techniques.

Correlation analysis of radiographic tooth width measurements obtained using the device-assisted and non-device techniques is summarized in Table 2. Both techniques demonstrated statistically significant correlations with the reference measurements (*p* < 0.0001). Scatter plots illustrating these relationships are shown in Figure 4.

### 3.3. Frequency of Radiographic Errors

A total of 196 intraoral radiographic exposures were analysed, comprising 98 device-assisted and 98 non-device images. The frequency and percentage distribution of radiographic errors are presented in Table 3. Cone cut, elongation, sliding occlusal plane deviation, apical cut, proximal overlap, and tooth-not-centred errors were recorded in both groups. Foreshortening was observed only in the non-device group (2/98; 2.04%), while blur was not observed in either group.

## 4. Discussion

This study evaluated the performance of a gyroscopic-guided, laser-assisted device designed to improve the geometric accuracy and reproducibility of intraoral radiography by reducing operator-dependent variability in cone positioning. Intraoral radiographs are particularly sensitive to small deviations in vertical and horizontal angulation, which can lead to elongation, foreshortening, overlap, and the need for repeat exposures [15]. The present findings demonstrate that device assistance is associated with improved agreement between measured and reference tooth dimensions and a lower frequency of common technical errors compared with the conventional free-hand bisecting-angle technique. It is important to note that the present study did not directly assess diagnostic accuracy or clinical diagnostic outcomes. The observed improvements relate specifically to geometric fidelity, measurement reproducibility, and reduction in selected technical radiographic errors. While improved geometric accuracy may indirectly support diagnostic reliability, no conclusions regarding diagnostic accuracy can be drawn from the current data.

For tooth height measurements, the stronger correlation and higher coefficient of determination observed with device assistance are consistent with the central role of vertical angulation in determining projected tooth height. Minor deviations in vertical angulation are known to result in elongation or foreshortening [16]. By providing gyroscopic stabilization and visual guidance, the device likely constrained unintended variations in vertical cone inclination. Similar improvements in measurement reproducibility have been reported with other alignment-assisting approaches, such as positioning lasers and guided cone systems, although these have largely focused on panoramic or limited intraoral applications [7,10].

Width measurements also demonstrated stronger correlations when the device was used, reflecting improved control of horizontal angulation. Mesiodistal tooth width is particularly susceptible to changes in horizontal beam orientation, which directly influence proximal overlap [8]. The present findings align with previous reports showing that free-hand intraoral radiography is prone to rotational errors around the vertical axis, especially among less experienced operators [3,17]. By maintaining a consistent beam trajectory, the gyroscopic device appears to reduce these lateral deviations.

Analysis of radiographic error patterns further supports the geometric benefits of device assistance. Cone-cut and elongation errors were less frequent in device-assisted images, consistent with improved beam centering and controlled vertical angulation [18]. Foreshortening was observed only in non-device images, which aligns with excessive vertical angulation, a known limitation of free-hand techniques [3]. Sliding occlusal plane errors occurred more frequently without device assistance, likely reflecting subtle yaw and roll deviations of the cone relative to the occlusal plane, a phenomenon also described in prior assessments of intraoral positioning errors [19,20].

Apical cut was the most frequent error in both techniques, although its occurrence was reduced with device use. Apical cut-off is commonly associated with incomplete receptor positioning or insufficient apical coverage by the central ray, particularly in posterior regions or anatomically constrained areas [21]. While the device cannot fully overcome patient- or anatomy-related limitations, its ability to stabilize beam orientation appears to mitigate technique-related contributors to this error. Similarly, reductions in proximal overlap and tooth-not-centred errors suggest improved horizontal alignment and more consistent targeting of the region of interest, both of which are critical for diagnostic completeness.

When considered in the context of existing literature, these findings extend prior work on alignment-assisting devices by demonstrating simultaneous improvements in linear measurement accuracy and error reduction using a single, cone-mounted gyroscopic system [6,7]. Unlike traditional positioning aids that primarily address receptor placement, the present device directly targets beam orientation, which remains a major source of variability even with digital sensors [11,12,13]. This distinction may explain the broad reduction in error categories observed in the present study.

### Comparison with Existing Laser-Guided Position Indicating Devices

Previous studies and commercially available laser position-indicating devices (PIDs) have primarily focused on assisting beam centering and receptor alignment by projecting static laser reference points or lines onto the patient’s face or sensor holder. These systems are typically designed to facilitate initial positioning but do not provide continuous feedback on cone angulation or dynamic correction of operator-induced deviations during positioning [7,22,23].

In contrast, the gyroscopic-guided device evaluated in the present study incorporates an inertial sensing component that continuously monitors cone orientation in real time. By integrating tri-axial gyroscopic feedback with laser-based visual guidance, the device provides dynamic information on both vertical and horizontal angulation, rather than relying solely on static beam-centering cues. This distinction allows the operator to identify and correct subtle angular deviations before exposure, which may contribute to the observed reduction in geometric distortion and selected radiographic errors.

Unlike commercially available laser PIDs that are often integrated into specific X-ray units or proprietary holders, the proposed device was designed as a lightweight, non-invasive add-on that can be attached to standard intraoral X-ray cones without modification of existing equipment. This modular design enhances adaptability across different radiographic systems and may reduce implementation barriers in routine clinical settings.

It should be noted that, similar to existing laser-assisted systems, the present device does not directly address errors related to receptor placement depth or anatomical constraints, as reflected by the persistence of apical cut errors. Therefore, while the gyroscopic-guided approach extends the functionality of conventional laser PIDs by adding angular stabilization, it should be viewed as complementary to, rather than a replacement for, established receptor positioning aids. Future comparative studies directly evaluating gyroscopic-guided devices against commercial laser PIDs under identical conditions are warranted to further define relative performance advantages.

The clinical relevance of this work lies in its potential to reduce operator-dependent variability in routine intraoral radiography, which remains a major source of image distortion, repeat exposures, and diagnostic inconsistency in daily practice. By providing real-time visual and angular feedback, the gyroscopic-guided device may support more consistent beam alignment, particularly for less experienced operators, such as dental interns, students, and newly qualified practitioners. Improved geometric fidelity of radiographs can facilitate more reliable assessment of root length, periapical status, bone levels, and interproximal contacts, which are critical for endodontic, periodontal, and restorative decision-making. Furthermore, by minimizing common technical errors that necessitate retakes, the device has the potential to reduce cumulative radiation exposure, improve workflow efficiency, and enhance patient comfort. Although the present study was conducted under phantom conditions, these findings provide a strong technical rationale for future clinical trials aimed at evaluating real-world diagnostic impact, learning curve benefits, and reductions in repeat imaging rates.

Several limitations should be acknowledged. A key limitation of the present study is that all radiographic acquisitions were performed on phantom models rather than on patients. While phantom-based testing allows strict control of geometric conditions and eliminates confounding variables such as patient movement, gag reflex, soft-tissue interference, and anatomical variability, it inherently limits external validity. Consequently, the findings reflect device performance under standardized conditions and may not fully represent clinical performance in patient-based settings. Nevertheless, phantom testing was intentionally selected as an essential preclinical step to isolate the technical effect of the gyroscopic-guided device and to establish baseline accuracy and error reduction prior to clinical application. Future studies involving patient cohorts are warranted to evaluate device performance under real-world clinical conditions.

A formal power calculation was not performed due to the pilot design; future clinical studies will be powered using effect sizes and variance estimates derived from the present dataset.

A potential limitation of this study is that all operators were dental graduates and general practitioners with baseline competency in intraoral radiographic techniques. While this selection reduced variability related to inexperience and ensured standardized image acquisition, it may limit generalizability to less experienced operators, such as undergraduate dental students or auxiliary staff. Consequently, the observed improvements associated with the device may represent conservative estimates, as greater relative benefit might be expected among operators with lower baseline proficiency. Future studies should evaluate device performance across a broader range of operator experience levels to better characterize its educational and clinical utility.

Future research should include clinical, patient-based studies with larger and more diverse operator groups to evaluate real-world applicability, learning curves, and potential reductions in repeat exposure rates. Integration of such guidance systems into routine clinical workflows may contribute to improved diagnostic consistency, enhanced radiation safety, and more standardized intraoral radiographic practice.

## 5. Conclusions

The gyroscopic-guided, laser-assisted radiographic device demonstrated an improvement in the geometric consistency of intraoral radiographs and a reduction in the frequency of several common technical errors when compared with the conventional free-hand bisecting-angle technique. These findings indicate enhanced control of beam angulation and improved reproducibility of linear measurements. However, the outcomes of this study reflect improvements in radiographic geometry and technical performance rather than direct diagnostic accuracy, as no clinical diagnostic endpoints were assessed. In addition, the persistence of apical cut errors despite device assistance suggests that challenges related to receptor positioning and anatomical constraints remain and warrant further refinement of device integration. Future patient-based studies are required to evaluate clinical performance and to determine whether improved geometric fidelity translates into measurable diagnostic benefits.

## Figures and Tables

**Table 1:** Pearson correlation statistics for tooth height measurements obtained using device-assisted and non-device techniques.

Height	Device	Non-Device
*N*	140	140
r-value	0.942	0.767
*p*-value	*p* < 0.0001	*p* < 0.0001
*p* value summary	***	***
Is the correlation significant? (alpha = 0.05)	Yes	Yes
R squared	0.887	0.589

Note: *** indicates *p* < 0.001.

**Table 2:** Pearson correlation statistics for tooth width measurements obtained using device-assisted and non-device techniques.

Width	Device	Non-Device
*N*	98	98
r-value	0.878	0.748
*p*-value	*p* < 0.0001	*p* < 0.0001
*p* value summary	***	***
Is the correlation significant? (alpha = 0.05)	Yes	Yes
R squared	0.770	0.560

Note: *** indicates *p* < 0.001.

**Table 3:** Frequency and percentage distribution of radiographic errors for device-assisted and non-device techniques (*N* = 98 per group).

Error Type	Error (Device)	% Error (Device)	Error (Non-Device)	% Error (Non-Device)	*p*-Value
CC (Cone Cut)	4	4.08%	14	14.29%	0.041 *
EL (Elongation)	6	6.12%	16	16.33%	0.008 **
FRSH (Foreshortening)	0	0.00%	2	2.04%	0.218
BLUR	0	0.00%	0	0.00%	–
SLPL (Sliding Occlusal Plane)	12	12.24%	26	26.53%	0.003 **
APCUT (Apical Cut)	37	37.76%	48	48.98%	0.002 **
PRXOLT (Proximal Overlap)	11	11.22%	26	26.53%	0.015 *
TNIC (Tooth Not in Center)	9	9.18%	20	20.41%	0.021 *

Note: * indicates statistically significant difference at *p* < 0.05; ** indicates statistically significant difference at *p* < 0.01.

## Data Availability

The data presented in this study are available from the corresponding author upon request. The data is not publicly available due to ethical restrictions.
